# Iodides for Scrofulous Children

**Published:** 1889-12-07

**Authors:** 


### IODIDES FOR SCROFULOUS CHILDREN.

M. E. Besnier recommends in cases in which it is desired to administer iodides for a long period the use of tincture of iodine or iodoform, in place of the iodides of the alkalies, as being in his experience better borne. He gives tr. iodi. 1m. daily in milk or arrowroot, or iodoform 0·005 milligr. $=\frac{1}{125}$ grain ter die.

